# In search of the ratio of miRNA expression as robust biomarkers for constructing stable diagnostic models among multi-center data

**DOI:** 10.3389/fgene.2024.1381917

**Published:** 2024-04-30

**Authors:** Cuidie Ma, Yonghao Zhang, Rui Ding, Han Chen, Xudong Wu, Lida Xu, Changyuan Yu

**Affiliations:** ^1^ College of Life Science and Technology, Beijing University of Chemical Technology, Beijing, China; ^2^ State Key Laboratory of Complex Severe and Rare Diseases, Department of Laboratory Medicine, Peking Union Medical College Hospital, Chinese Academy of Medical Science and Peking Union Medical College, Beijing, China; ^3^ Shenyang Medical College, Shenyang, China; ^4^ Beijing Hotgen Biotech Co., Ltd., Beijing, China

**Keywords:** biomarker, microRNA interactions, batch effect, multi-center data, disease classifications

## Abstract

MicroRNAs (miRNAs) are promising biomarkers for the early detection of disease, and many miRNA-based diagnostic models have been constructed to distinguish patients and healthy individuals. To thoroughly utilize the miRNA-profiling data across different sequencing platforms or multiple centers, the models accounting the batch effects were demanded for the generalization of medical application. We conducted transcription factor (TF)-mediated miRNA–miRNA interaction network analysis and adopted the within-sample expression ratios of miRNA pairs as predictive markers. The ratio of the expression values between each miRNA pair turned out to be stable across multiple data sources. A genetic algorithm-based classifier was constructed to quantify risk scores of the probability of disease and discriminate disease states from normal states in discovery, with a validation dataset for COVID-19, renal cell carcinoma, and lung adenocarcinoma. The predictive models based on the expression ratio of interacting miRNA pairs demonstrated good performances in the discovery and validation datasets, and the classifier may be used accurately for the early detection of disease.

## 1 Introduction

MicroRNAs (miRNAs) have emerged as valuable biomarkers for the early detection of diseases due to their tissue-specific expression profiles (Chen et al., 2008; Song et al., 2023; Lv and Sun, 2024). However, the measure of miRNA expression levels may vary across different platforms or protocols, which limits the application of diagnostic models. This problem is known as batch variance and is prevalent in cross-sequencing platforms (Lazar et al., 2013) and multi-center data (Leek et al., 2010; Heinicke et al., 2020a; Heinicke et al., 2020b; Ibing et al., 2021). The difference in data distribution may be an obstacle to obtain reliable conclusions in the joint analysis of multiple center datasets, and it prevents the cross-validation of models on external datasets (Leek et al., 2010; Zhang et al., 2021; Whalen et al., 2022; Peng et al., 2024). Thus, the effective handling of batch effects is the first problem that needs to be solved in the integrative analysis of large-scale biological datasets (Goh et al., 2017).

Several batch effect correction methods have been developed to facilitate the joint analysis of multi-center data. The “ComBat-seq” tool based on the negative binomial regression model was developed specifically for RNA-seq count data (Zhang et al., 2020a). The “removeBatchEffect” function in the “limma” package can be used to correct data variation based on the linear regression model (Ritchie et al., 2015). However, these correction methods require the artificial transformation of data shapes, which may introduce false discoveries (Nygaard et al., 2016). In contrast, the intrinsic regulatory networks are less likely to be affected by different sequencing protocols, and the pathway-derived genes show potential to be a type of normalizer-free and batch-insensitive markers. Under this consideration, we propose a promising novel tool to integrate datasets from multiple sources, termed as the ratio of the expression values between related miRNAs (ERRmiR), by calculating the ratio of expression values of two related miRNAs in the intrinsic regulatory networks.

The miRNA interaction network was constructed based on prior knowledge to discover ERRmiR features with a biological significance. It is widely known that miRNAs not only regulate the expression of protein-coding mRNAs but also target non-coding RNAs, including long non-coding RNAs and miRNAs (Hill and Tran, 2018; Vishnubalaji et al., 2022; Shang et al., 2023). The miRNAs can directly bind to the 3′UTR of transcription factors (TFs), which can also reverse activate or repress miRNA expressions (Vishnubalaji et al., 2022; Khotib et al., 2023). For example, miR-181b affects the expression of miR-21 through the TF FOS, a critical signaling protein for glioma progression (Tao et al., 2013); miR-660-5p controls the expression of miR-486-5p via mouse double minute 2 (MDM2) and p53 (also known as TP53) in a study of lung cancer (Borzi et al., 2017). A recent review summarizes numerous examples of miRNA–>TF (TF regulated by miRNAs) and TF–>miRNA (miRNAs regulated by TF) interactions in various cancers, demonstrating the importance of the interaction between miRNA and pluripotent TFs in determining the occurrence of human cancers (Vishnubalaji et al., 2022). All these examples provide important clues for understanding the role of the TF-mediated miRNA functional network in tumor regulation.

In this study, we constructed a TF-mediated miRNA interaction network using public databases and demonstrated that the ERRmiR features were relatively insensitive to batch effects in multi-center studies. We then adopted a genetic algorithm in the feature screening process to avoid the dimension curse, which had a great capacity for selecting markers with stable performances in developing diagnostic models. Lastly, we used three independent examples involving plasma and tissue samples to investigate the predictive performance.

## 2 Materials and methods

### 2.1 Construction of the miRNA interaction network

The TF-mediated miRNA–miRNA interaction network was constructed by combining the data of miRNA–>TF and TF–>miRNA relationships. If miRNA_a regulated a TF that was regulated by miRNA_b, miRNA_a was assumed to be able to influence miRNA_b, and they were connected in the miRNA interaction network.

The regulatory network datasets were collected from several public databases. The experiment validated that microRNA–target pairs were collected from miRTarBase (Huang et al., 2020), among which 8,014 targets were recognized as TFs based on the hTFtarget (Zhang et al., 2020b) and AnimalTFDB (Hu et al., 2019) databases. The 1,266 records of TF-regulating precursor miRNAs were obtained from the TransmiR v2.0 database (Tong et al., 2019). Combining the miRNAs–>TFs and TFs–>miRNA datasets (here, –> denotes a regulatory relationship), a total of 51,770 pre-miRNA indirect interactions were obtained, and then, pre-miRNAs were mapped to mature miRNAs according to the miRBase genomic coordinates. Finally, the miRNA–miRNA interaction network was constructed based on the 75,507 unique records of the indirect interaction relationships.

### 2.2 Predictive feature generation

The features were generated by calculating the expression ratio for each miRNA pair in the reconstructed miRNA interaction network. miRNAs with an expression value smaller than 100 were filtered out to ensure stable detection. The feature constructed with the connected pair of miRNA_a and miRNA_b was denoted by ERRmiR (a,b) and calculated as follows:

$$ERRmiR\left(a,b\right)=\frac{{Expression\;of\;miRNA}_{a}}{{Expression\;of\;miRNA}_{b}+1},$$

where the denominator was added by 1 to avoid the divisor being 0.

### 2.3 Data collection and pre-processing

The robustness of ERRmiR features was investigated on the datasets using different library preparation kits [GSE133719 and GSE141658 datasets on the Gene Expression Omnibus (GEO) (Clough and Barrett, 2016) database], and then, datasets for three different disease categories were collected to construct the predictive models, namely, COVID-19, renal cell carcinoma (RCC), and lung adenocarcinoma (LUAD) projects [the NCI’s Genomic Data Commons (GDC) (Jensen et al., 2017) database and GEO; Table1]. We searched the GEO database to identify datasets meeting the following criteria: both disease and control groups had sample sizes of 10 or more, and each disease had at least 2 datasets with consistent sample types and sequencing platforms. From the filtered datasets, representative validation sets were chosen, which included viral respiratory infections and cancers caused by non-viral mechanisms. These sets were selected to ensure diversity in etiology, validation centers, and sample types, thereby ensuring the generalizability of our feature selection model across different populations. The miRNA expression matrices in the CPTAC (Edwards et al., 2015)/TCGA (Hutter and Zenklusen, 2018) database were downloaded using the GDC tool, and the annotation and quantification were performed using exceRpt (Rozowsky et al., 2019) to obtain the expression matrices of miRNAs. For comparing the results among different datasets, counts of reads were uniformly converted to reads per million (RPM) mapped read values. In the COVID-19 project, the plasma of persons with non-severe symptoms (mild patients and healthy) was categorized as the controls, and the plasma of those with serious symptoms was used as the disease samples. In the RCC and LUAD projects, normal tissues were categorized as the controls, and primary tumor tissues were used as the disease samples.

**TABLE 1:** Sample information in three projects. The datasets with the most samples were selected as discovery (lines colored by gray background), and the other datasets were used as validation (lines colored by white background) for each project.

Project	Dataset	Number of control cases	Number of disease cases	Platform	Source
COVID-19	GSE178246^a^	272	264	Illumina NextSeq 500	Plasma
	GSE176498	29	16	Illumina NextSeq 550	Plasma
RCC	CPTAC-3-RCC	148	311	Illumina	Tissue
	TCGA-KIRP	34	34	Illumina HiSeq 2000	Tissue
	GSE109368	12	12	Illumina NextSeq 500	Tissue
LUAD	CPTAC-3-LUAD	102	111	Illumina	Tissue
	GSE110907	48	48	Illumina HiSeq 2000	Tissue
	GSE196633	10	10	Illumina HiSeq 2500	Tissue

^a^ Each sample has four pieces of sequencing data for GSE178246 and treated as four cases.

### 2.4 Feature screening and classification modeling

In each project, the dataset with the most samples was used as the discovery dataset and divided into a training set and a test set proportionally, i.e., 0.75:0.25. In the training dataset, the univariate analysis of the ERRmiR features was performed, the expression fold change in disease samples against the controls and the fdr-adjust *p*-value were obtained. The “sklearn-genetic” package was adopted to screen the optimal subsets of features. The features with higher appearance frequencies in the optimal subsets were selected as targets for the disease.

The “scikit-learn” package was used to build models for disease classifications. The learning curves were used to detect whether the estimator was overfitting during model training. The trained model was validated on a test set and the external validation datasets for each project.

### 2.5 Statistical analysis and visualization

The quartile plots of miRNA expression/ERRmiR feature values were drawn using the Matplotlib tool. The *p*-values and fdr-corrected q-values were calculated using SciPy. The miRNA network was visualized using Pyvis and seaborn tools. In miRNA pathway enrichment analyses, target genes of miRNAs were first identified through the TarBase database using the multMiR package in R language, and then, pathway enrichments were performed using clusterProfiler.

## 3 Results

### 3.1 The schematic of ERRmiR signature generation and screening

**FIGURE 1:**
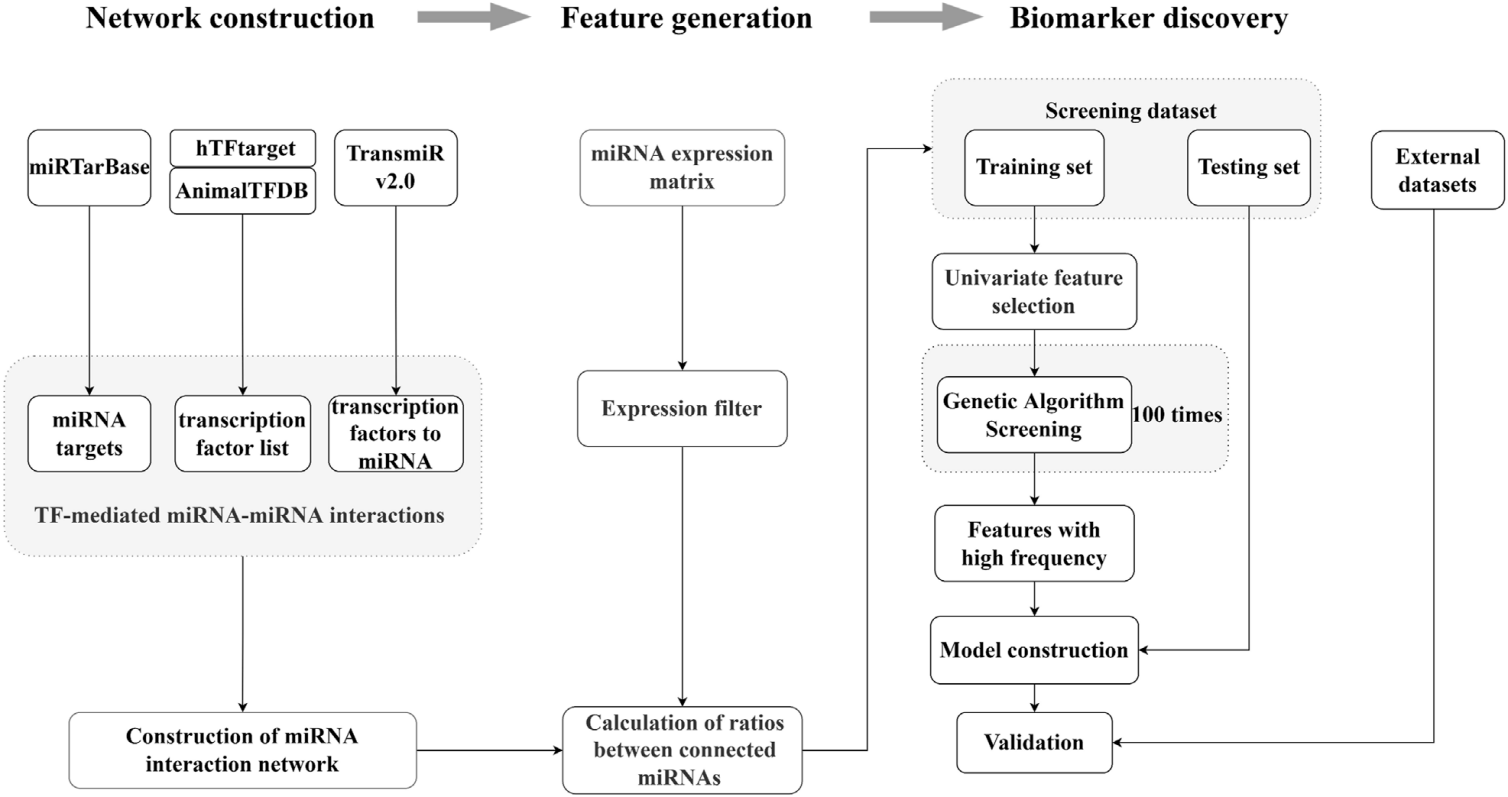
Overview of the ERRmiR marker discovery process. The miRNA network was constructed based on the transcription factor (TF)-mediated interactions, and the ERRmiR features were calculated between the connected genes in the network. Target screening and model construction were performed based on the ERRmiR features of the screening dataset and verified on the validation dataset.

We developed a screening method for the generation of the ERRmiR signature based on machine learning (Figure 1). We first constructed the miRNA interaction network by integrating several databases, including miRTarBase, hTFtarget, AnimalTFDB, and TransmiR v2.0. We then calculated the expression ratios of related miRNA pairs as ERRmiR features. The discovery dataset was randomly divided into training and test sets, and the features were filtered in the training set using univariate analyses according to the fold change of the mean expressions between two groups. We used a genetic algorithm to screen the features, and those with higher frequencies in the screening processes were selected as candidate markers. The trained model was validated on the test set within the same screening dataset and evaluated on external validation datasets. This approach was suitable for discovering biomarkers for various samples.

### 3.2 Construction of the miRNA interaction network

**FIGURE 2:**
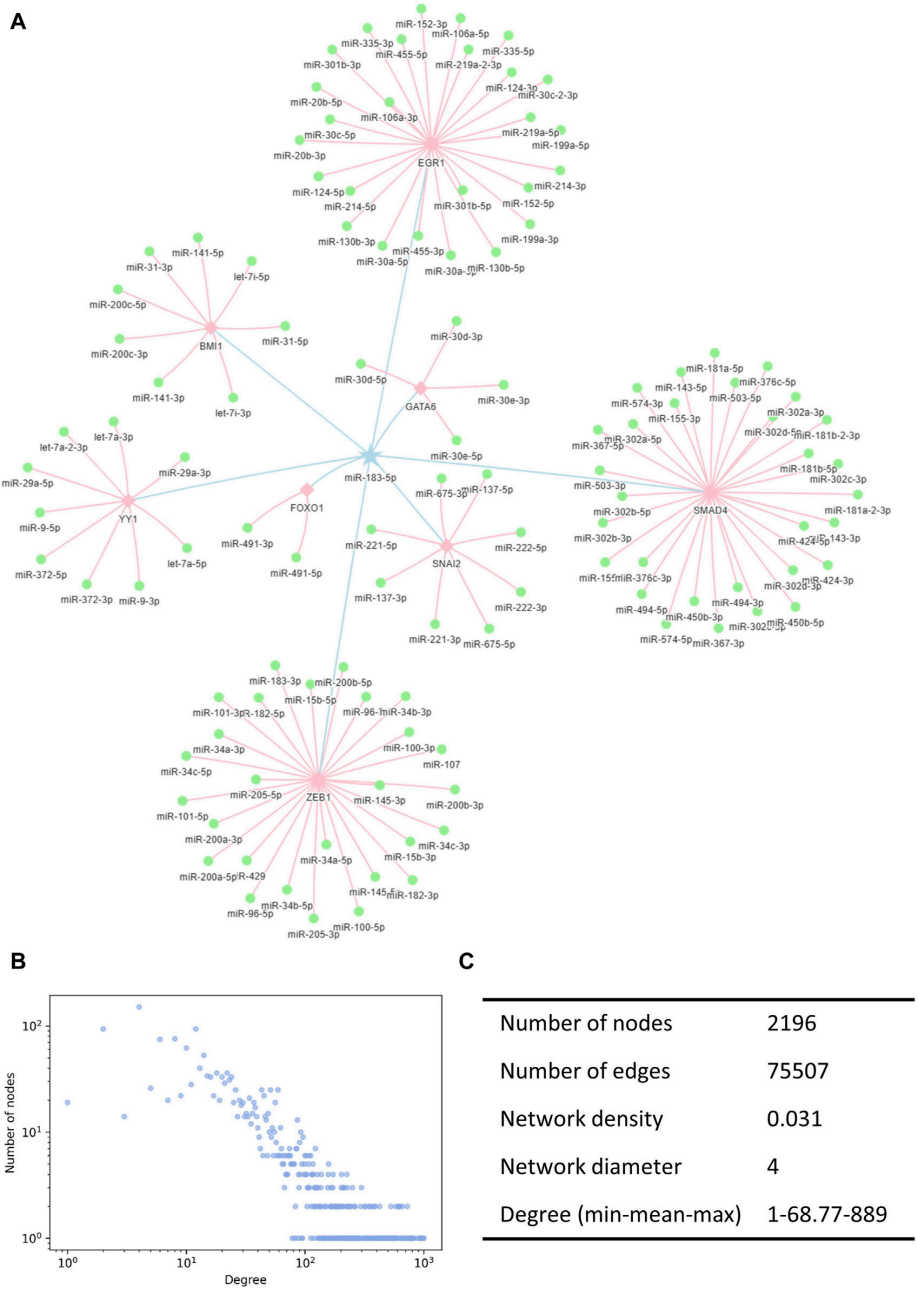
Illustration of the miRNA interaction network. **(A)** TF-mediated miRNA–miRNA indirect interactions. Pentagrams denote the regulating miRNAs, squares denote the TFs, and circles denote the regulated miRNAs. **(B)** Degree distribution of the miRNA interaction network followed a power-law tail. **(C)** Topological characteristics of the interaction network. TF, transcription factors.

We constructed the miRNA interaction network based on indirect interactions mediated by TFs. The interactions indicated that the expression of one miRNA induced the activation or inhibition of other miRNAs. miR-183-5p is taken as an example to show how miRNAs regulate other miRNAs through TFs (Figure 2A). Here, the pentagram-labeled miR-183-5p is a regulatory miRNA, which regulates the square-labeled TF and further affects the round-labeled target miRNAs. The blue linkages represented the interaction of miR-183-5p acting on the TF, and the pink linkages represented the effects of TFs on other miRNAs. The complete miRNA–miRNA interaction network contained 75,507 unique records of indirect interaction relationships among 2,196 miRNAs (Supplementary Table S1). The degree distribution and topological parameters indicated that the miRNA interaction network has canonical scale-free and small-word characteristics (Figures 2B, C).

### 3.3 Characterization of ERRmiR signatures

**FIGURE 3:**
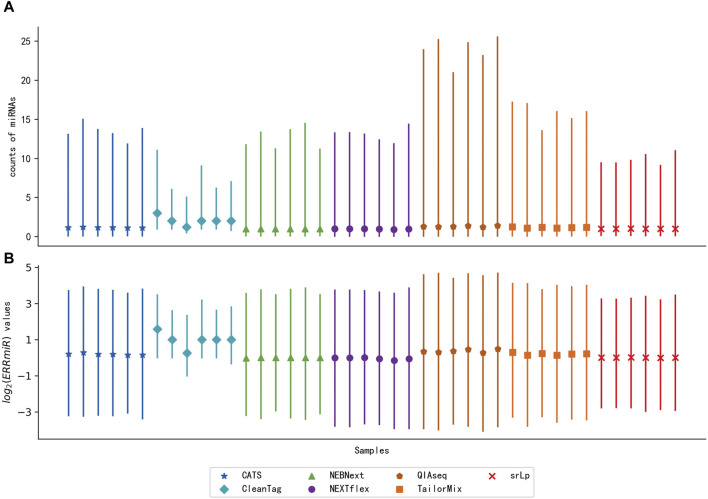
Quartile plots of miRNA expression **(A)** and log2 ratios of every two miRNAs **(B)** for each sample. Each plot was represented with the median (a solid point), the 0.25 quartile, and the 0.75 quartile of the distribution.

To verify the hypothesis that the expression ratios between the interacting miRNAs would be stable across multi-center data, the distribution of ERRmiR values was compared with the distribution of the miRNA expression levels of the same samples (Figure 3). The sequencing datasets of the peripheral blood CD8^+^ T cells in triplicate from rheumatoid arthritis (RA) patients and healthy controls were generated by different library construction methods. The quartile plots showed that the original miRNA expression data generated by different library preparation kits had significant variance on the scale and distributions (Figure 3A), while the variation of ERRmiR features decreased (Figure 3B), which demonstrated the potential of ERRmiR features as batch-insensitive markers. We presented three application examples from various sample types and diseases.

### 3.4 Prediction of COVID-19 patients with severe symptoms using plasma ERRmiR signatures

**FIGURE 4:**
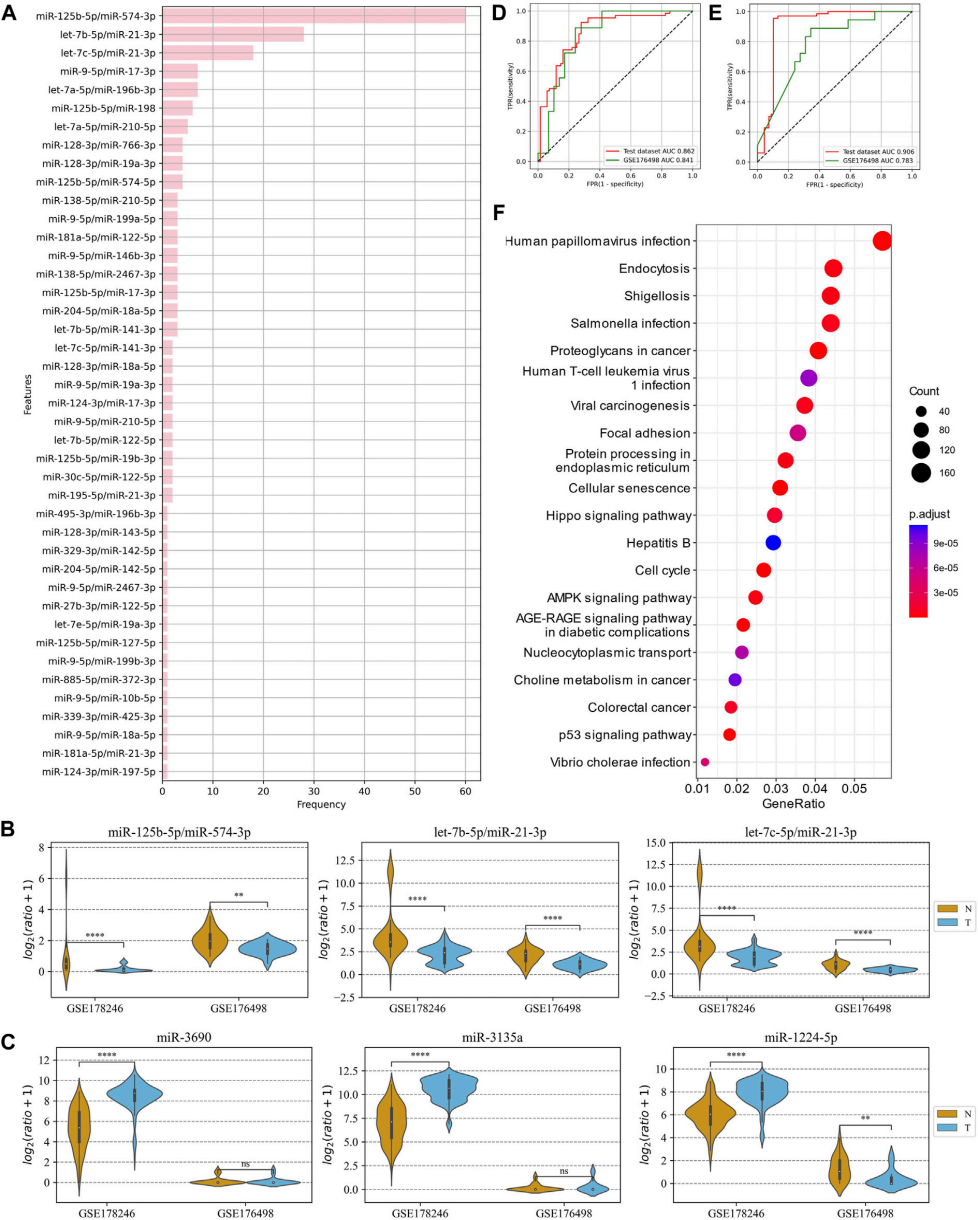
Analysis of ERRmiR features in the COVID-19 project. **(A)** Occurrence frequencies of the ERRmiR features in the 100-time genetic algorithm. **(B,C)** The top three high-frequency ERRmiRs showed a relatively stable regulatory trend in both datasets rather than miRNAs. ROC curves of the models based on ERRmiR markers **(D)** and miRNA markers **(E)**. **(F)** Pathway enrichment analysis of miRNAs involved in ERRmiR markers showed the top 20 enriched pathways.

The advantage of ERRmiR features was first investigated on the dataset of COVID-19 plasma samples. GSE178246 was randomly divided into a training and test set, and GSE176498 was used as the external validation set. The 42 ERRmiR features were obtained by conducting the genetic algorithm 100 times on the screening dataset. As shown in Figure 4A, the frequency distribution of the ERRmiR appearance was very steep, 3 ERRmiRs have frequencies greater than 10, and the highest frequency was up to 60. We selected the top three high-frequency features as markers and tested them on the validation set. As expected, they were significantly different between the serious and non-serious groups (*p* < 0.05) and showed consistent trends across multiple datasets (Figure 4B; Supplementary Table S2). The TFs intervening between the ERRmiR pairs for the top three high-frequency features were mothers against decapentaplegic homolog 4 (SMAD4), PR domain zinc finger protein 1 (PRDM1), and forkhead box O3 (FOXO3). To confirm the batch-insensitive nature of the ERRmiR features, the biomarker selection was also directly applied on the expression matrix of miRNAs. As shown in Figure 4C, the targets screened from the expression matrix of miRNAs lost effectiveness across the batches of data, with miR-1224-5p even showing opposite regulation trends (Supplementary Table S3). Based on the three high-frequency ERRmiR markers, the C-support vector classification (SVC) model that was established on the training set showed stable high performances on both the test set and validation dataset (Figure 4D). The model with three high-frequency miRNA panels had a high area under curve (AUC) of 0.906 on the test set but failed on the independent validation set with an AUC of 0.783 (Figure 4E). The performance verification data of the model are shown in Table 2. In addition, the five miRNAs of three ERRmiR markers were used for pathway enrichment (Figure 4F). Infection pathways of bacteria and viruses, including *Salmonella* infection and human papillomavirus infection, were significantly enriched.

**TABLE 2:** Comparison of miRNAs and ratio feature model performance on the COVID-19 data.

Signature	Cohort	Sensitivity	Specificity	AUC
miRNA	Test	0.955	0.897	0.906
	GSE176498	0.889	0.655	0.783
ERRmiR	Test	0.924	0.721	0.862
	GSE176498	0.889	0.759	0.841

### 3.5 Prediction of the renal cell carcinoma using tissue ERRmiR signatures

**FIGURE 5:**
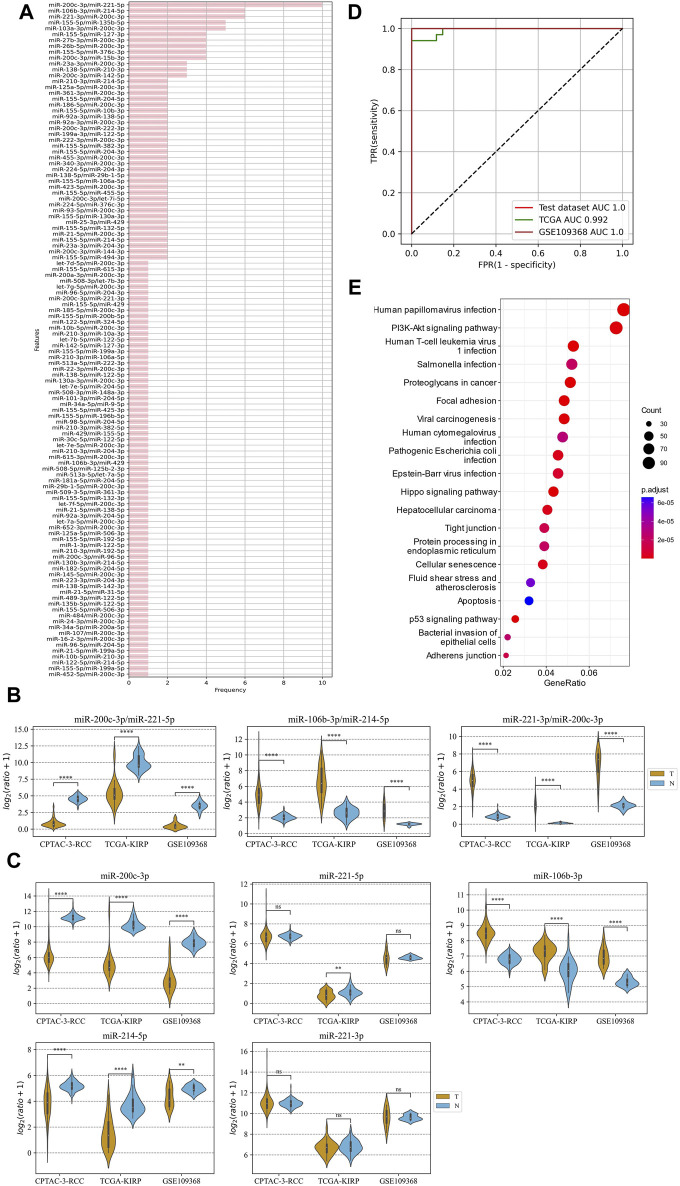
ERRmiR markers discovered in the renal cell carcinoma (RCC) project. **(A)** The frequency distribution of the ERRmiR features. Violin plots of the top three high-frequency ERRmiR features **(B)** and the composite miRNAs **(C)** among the three independent datasets. **(D)** ROC curves of the model based on the ERRmiR markers. **(E)** Pathway enrichment analysis of miRNAs in the ERRmiR markers.

The method of marker discovery was also validated on the dataset of the RCC tissue samples. The CPTAC-RCC dataset was used for screening targets and building the model, and TCGA-KIRP and GSE109368 datasets were used for external validations. After conducting the genetic algorithm, we obtained 115 miRNA pairs (Figure 5A). We take the top three highest frequent ERRmiR features as biomarkers, which showed significant differences between the cancer and control groups (*p* < 0.05), with consistent regulation trends across multiple datasets (Figure 5B; Supplementary Table S4). The TFs intervening between the ERRmiR pairs for the top three high-frequency features were Jun proto-oncogene (JUN, AP-1 transcription factor subunit), HIF1A (HIF1A), nuclear factor erythroid 2-related factor 2 (NFE2L2), and zinc finger E-box-binding homeobox 2 (ZEB2). As part of the miRNAs in ERRmiR markers, miR-221-3p and miR-221-5p were not significantly differentially expressed between the two sample groups in all the datasets (Figure 5C; Supplementary Table S5). A prediction model using the SVC algorithm was established on the training dataset and achieved high AUC values on both independent validation datasets (Figure 5D). The performance verification data of the model are shown in Table 3. The five miRNAs comprising the three ERRmiR markers were significantly enriched in several pathways associated with cancers (Figure 5E). In particular, the p53 signaling pathway and Hippo signaling pathway had been widely reported to be associated with RCC (Gurova et al., 2004; Guan et al., 2018).

**TABLE 3:** Model performance on the renal cell carcinoma (RCC) data.

Cohort	Sensitivity	Specificity	AUC
Test	1.000	1.000	1.000
TCGA	0.941	1.000	0.992
GSE109368	1.000	1.000	1.000

### 3.6 Prediction of lung adenocarcinoma using tissue ERRmiR signatures

**FIGURE 6:**
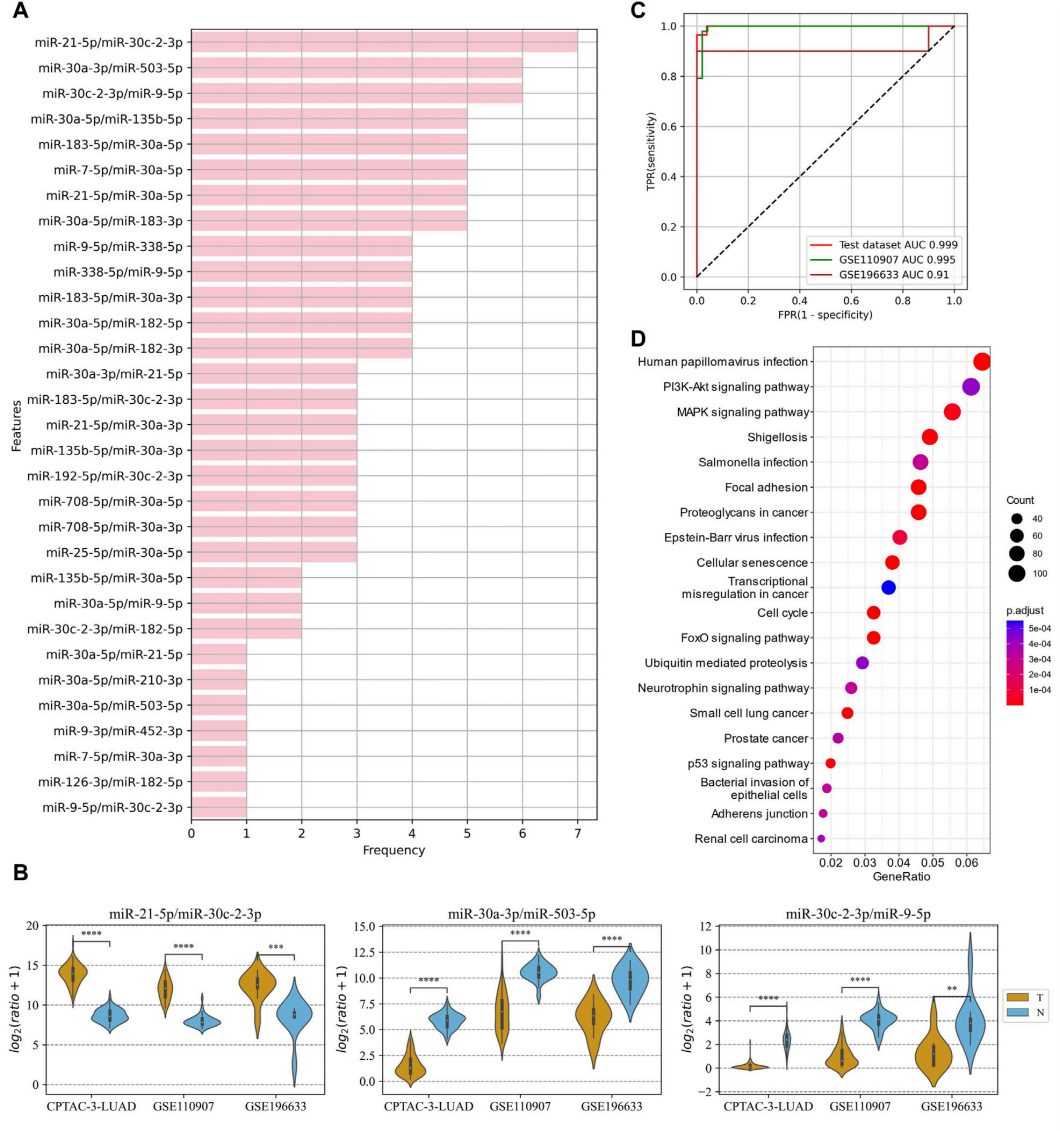
Discovery results on the lung adenocarcinoma (LUAD) project. **(A)** Statistics of the frequencies of ERRmiR features. **(B)** Violin plots of ERRmiR features ranked top three by frequency. **(C)** ROC curves of the models based on ERRmiR markers. **(D)** Pathway enrichment analysis of miRNAs involved in the ERRmiR markers.

In the LUAD project, the CPTAC-LUAD dataset was used to screen the ERRmiR features and build the model, and the GSE110907 and GSE196633 datasets were used for external validations. Thirty one ERRmiRs were obtained by conducting the genetic algorithm with a relatively flat frequency distribution, as shown in Figure 6A. We selected the top three highest frequent ERRmiR features as markers, which presented consistent trends of significant differences between the cancer and control groups (*p* < 0.05) across multiple datasets (Figure 6B; Supplementary Table S6). The TFs intervening between the ERRmiR pairs for the top three high-frequency features were nuclear factor kappa B (NFKB1), myocyte enhancer factor 2A (MEF2A), and Yin Yang 1 (YY1). The model constructed in the training set had AUC values of 0.995 and 0.91 in the GSE110907 and GSE196633 validation sets, respecively (Figure 6C). The performance verification data of the model are shown in Table 4. The five miRNAs of the three ERRmiR markers were significantly enriched in the p53 signaling, cell cycle, and PI3K-Akt pathways, which are widely reported to be associated with LUAD (Huang et al., 2022; Tang et al., 2022; Zhang et al., 2022) (Figure 6D).

**TABLE 4:** Model performance on the lung adenocarcinoma (LUAD) data.

Cohort	Sensitivity	Specificity	AUC
Test	0.964	1.000	0.999
GSE110907	1.000	0.958	0.995
GSE175462	0.900	1.000	0.910

## 4 Discussion and conclusion

The miRNA biomarkers have shown initial success in disease diagnosis and prognosis monitoring (Inoue and Inazawa, 2021); however, different sequencing prepares can cause variances across different batches, making it difficult to use the normalization of expression matrices alone for multi-center applications. In this study, we included three types of miRNA– miRNA interactions (direct interactions, indirect interactions, and global interactions) summarized in a previous review (Hill and Tran, 2021) and considered the indirect miRNA interactions mediated by TFs. Coordinated with an integrated screening method utilizing the genetic algorithm, we demonstrated the effectiveness of this strategy at tissue and plasma levels in three datasets and demonstrated its capacity for universal usage in developing diagnostic and classification models. The generalizability of our findings across diverse datasets was demonstrated through validation in multiple datasets from various sources, encompassing different diseases such as COVID-19, RCC, and LUAD. Specifically, our approach has been validated in two distinct datasets for COVID-19, three separate datasets for RCC, and three different datasets for LUAD. This multi-dataset validation strategy enhances the robustness and reliability of our findings by confirming the consistency and effectiveness of our method across different disease types and data sources.

Biomedical big data are often described as “small sample size in high-dimensional space,” indicating a scenario where the sample size is small but each individual possesses a high-dimensional feature set (Zeng et al., 2016). High-dimensional data often lead to the curse of dimensionality, resulting in less reliable predictive analysis. Feature selection is a crucial step in classification tasks as retaining important features through feature selection can expedite the training process and address the curse of dimensionality. Genetic algorithms can be utilized to select the optimal subset of candidate features through crossover and mutation operations. As an embedded feature selection method, its feature selection process requires integration with machine learning models, such as the SVC model selected in this study. Tournament selection, the most popular selection strategy in genetic algorithms, was chosen in this study due to its advantages including lower complexity, less susceptibility to local optima, and ease of parallelization compared to other selection methods like roulette wheel selection (Shukla et al., 2015).

The miRNA possesses various mechanisms to regulate its generation and expression. In addition to directly targeting primary or precursor miRNA to modulate miRNA generation, it can also regulate the expression of TF–mRNA by directly binding to its 3′UTR. Simultaneously, TFs can induce or inhibit miRNA expression (Zhang et al., 2020a; Vishnubalaji et al., 2022). Moreover, many miRNAs may synergistically drive molecular changes, resulting in greater effects than individual miRNAs alone (Bertero et al., 2014). Among these interactions, the miRNA–>TF–>miRNA interaction represents one significant mechanism, as two TF-mediated miRNAs may involve multiple related TFs, and parts of miRNAs are influenced by more than one miRNA. The feature selection method employed in this study can identify robust differential miRNA features relevant to dataset diseases from numerous interactions, aiding in the more accurate identification of potential biological mechanisms and disease-related molecular biomarkers.

Previous studies have also explored the use of target ratios as features. Research has investigated the creation of novel features based on the relative expression order of genes within samples, achieving insensitivity to batch effects and facilitating the development of diagnostic models across different platforms, including sequencing and microarray data (Yang et al., 2020). Furthermore, studies have employed microarray data from two cohorts to generate new features based on the size relationship of miRNA pairs, converting quantitative data into qualitative data via a binarization process, albeit leading to notable information loss (Liu et al., 2021). Researchers identified 93 miRNAs showing significant differential expression among healthy controls and adenoma and colorectal cancer groups. They computed ratios between these 93 miRNAs in all possible pairwise combinations, resulting in 2,529 ratios. Among these, 36 miRNA ratios were found to exhibit significant differences in colorectal cancer samples compared to healthy controls and adenoma samples (Zhang et al., 2018a). The combination of two negatively correlated miRNAs may offer substantial potential for distinguishing experimental groups. Differential expression miRNA pairs were derived through the subtraction of the original Ct value of one miRNA from the Ct value of another miRNA, providing a novel avenue for biomarker discovery through self-normalization (Matthaei et al., 2012). Previous studies on miRNA ratio features have not systematically explored all miRNAs, whether by generating ratio features first and then screening for differences or by initially calculating differences in miRNAs and subsequently deriving ratios. Some studies validate only within different batches from the same center, without validating samples from different centers. Furthermore, these studies have often overlooked the potential biological significance inherent in these ratios, particularly miRNA–miRNA interactions. Our feature selection method is robust, effectively mitigates batch effects, and applicable across datasets sourced from diverse centers. Moreover, it has the ability to forecast biologically pertinent miRNA pairs, thereby establishing the groundwork for acquiring robust and high-performing biomarkers.

Using this protocol, we discovered some miRNAs with biological significance in all three examples. let-7b-5p, which is a selected marker for predicting severe COVID-19, plays a role in regulating ACE2 and DPP4 receptors and is significantly downregulated in nasopharyngeal swabs of patients (Latini et al., 2022); miR-21-3p, which is regulated by let-7b-5p, shows an upregulation trend in this project and is consistent with the previous experiments of mice infected with COVID-19 (Nersisyan et al., 2020). miRNA-mediated transcription factors are closely associated with COVID-19. Modulating the SMAD signaling pathway to enhance Robo4 expression holds promise in alleviating vascular permeability and mortality in COVID-19 (Cheema et al., 2021). FOXO transcription factors play crucial roles in maintaining normal cellular physiology by regulating survival, apoptosis, oxidative stress, and the development and maturation of T and B lymphocytes. The activation of FOXO can be utilized as a strategy to mitigate inflammatory outbreaks following SARS-CoV-2 infection (Morita et al., 2023).

The ERRmiR marker selected in the RCC project is found to be a critical oncogene in previous studies. The high expression of miR-106b-3p may be an important factor in predicting poor prognosis in RCC patients (Li et al., 2016; Liu et al., 2019), and the overexpression of miR-214-5p attenuates cell proliferation and metastasis (Guo et al., 2021). Upregulation of miR-200c-3p inhibits proliferation, migration, and invasion and induces apoptosis in RCC cells (Li et al., 2019). miRNA-mediated transcription factors are closely associated with RCC. HIF1A is upregulated in RCC tissues and closely correlated with tumor size and differentiation (Chen et al., 2021). The positive expression of ZEB1 is associated with poor prognosis in RCC patients (Harb et al., 2018).

In the LUAD project, the pairs containing miR-30a-3p or miR-30c-2-3p have been screened out. The role of the miR-30 family as tumor suppressors has been validated in previous reports (Saleh et al., 2019); in particular, miR-30c-2-3p is reported to inhibit tumor progression in esophageal squamous cell carcinoma, breast cancer, and hepatocellular carcinoma (Zhang et al., 2018b; Ma et al., 2018; Zhang et al., 2019). miR-9-5p and miR-503-5p, which are related with miR-30 in the ERRmiR markers, have also been reported to be associated with cell proliferation, migration, and invasion in non-small cell lung cancer (Sun et al., 2017; Zhu et al., 2021). miRNA-mediated transcription factors are closely associated with lung cancer. The expression of NFKB is related to the tumor stage, lymph node metastasis, and 5-year survival rate in lung cancer (Zhang et al., 2023). YY1 is upregulated in lung cancer tissues, and its higher expression correlates with larger tumor size, poor differentiation, higher TNM stage, and lymph node metastasis. The ectopic expression of YY1 in lung cancer cells promotes cell proliferation and invasion, while YY1 silencing suppresses cell proliferation and induces apoptosis (Huang et al., 2017; Zhu et al., 2023). These miRNAs and TFs are disease-related and have been validated in previous studies. The results further demonstrate that the proposed approach in this study is more helpful in exploring the pathogenic mechanisms of diseases.

We propose an algorithm based on the expression ratio of interacting miRNAs for feature selection. The features selected by this method are stable, capable of removing batch effects, and contribute to data standardization and consistency, providing a basis for obtaining high-performing stable biomarkers. Moreover, our method can identify biologically relevant miRNA pairs, further deepening the understanding of disease pathogenesis. The algorithm relies on prior knowledge about miRNA interactions, effectively reducing the dimensionality of features, alleviating the pressure of feature selection, and facilitating the discovery of true relationship markers. Although we validated the feasibility of our feature selection method across datasets from three different diseases, our study has certain limitations. It is confined to the same detection platform of one disease. We did not investigate the impact of platform transition on differential targets, such as the stability of differential targets between NGS and QPCR platforms. Therefore, further consideration of platform migration is necessary to ensure the robustness and applicability of our method across diverse settings. We will continue our efforts to address these limitations through rigorous validation and method refinement to fully realize its potential in clinical practice.

In conclusion, this study introduces an innovative feature selection algorithm based on the expression ratio of interacting miRNAs. Leveraging prior knowledge about miRNA interactions, the algorithm effectively reduces feature dimensionality, easing the burden of feature selection and aiding in the discovery of genuine relationship markers. This approach not only ensures stable feature selection, eliminating batch effects and facilitating data standardization and consistency, but also identifies biologically relevant miRNA pairs, thereby enhancing our understanding of disease pathogenesis. This method lies in providing a robust tool for the discovery of stable and biologically relevant biomarkers, offering new avenues and methodologies for early disease diagnosis and treatment.

## Data Availability

The datasets presented in this study can be found in online repositories. The names of the repository/repositories and accession number(s) can be found in the article/Supplementary Material.

## References

[B1] Bertero T. Lu Y. Annis S. Hale A. Bhat B. Saggar R. (2014). Systems-level regulation of microRNA networks by miR-130/301 promotes pulmonary hypertension. J. Clin. Invest. 124 (8), 3514–3528. 10.1172/JCI74773 24960162 PMC4109523

[B2] Borzi C. Calzolari L. Centonze G. Milione M. Sozzi G. Fortunato O. (2017). mir-660-p53-mir-486 network: a new key regulatory pathway in lung tumorigenesis. Int. J. Mol. Sci. 18 (1), 222. 10.3390/ijms18010222 28124991 PMC5297851

[B3] Cheema P. S. Nandi D. Nag A. (2021). Exploring the therapeutic potential of forkhead box O for outfoxing COVID-19. Open Biol. 11 (6), 210069. 10.1098/rsob.210069 34102081 PMC8187014

[B4] Chen M. Wei X. Shi X. Lu L. Zhang G. Huang Y. (2021). LncRNA HIF1A-AS2 accelerates malignant phenotypes of renal carcinoma by modulating miR-30a-5p/SOX4 axis as a ceRNA. Cancer Biol. Med. 18 (2), 587–603. 10.20892/j.issn.2095-3941.2020.0209 33710813 PMC8185866

[B5] Chen X. Ba Y. Ma L. Cai X. Yin Y. Wang K. (2008). Characterization of microRNAs in serum: a novel class of biomarkers for diagnosis of cancer and other diseases. Cell Res. 18 (10), 997–1006. 10.1038/cr.2008.282 18766170

[B6] Clough E. Barrett T. (2016). The gene expression Omnibus database. Methods Mol. Biol. 1418, 93–110. 10.1007/978-1-4939-3578-9_5 27008011 PMC4944384

[B7] Edwards N. J. Oberti M. Thangudu R. R. Cai S. McGarvey P. B. Jacob S. (2015). The CPTAC data portal: a resource for cancer proteomics research. J. Proteome Res. 14 (6), 2707–2713. 10.1021/pr501254j 25873244

[B8] Goh W. W. B. Wang W. Wong L. (2017). Why batch effects matter in omics data, and how to avoid them. Trends Biotechnol. 35 (6), 498–507. 10.1016/j.tibtech.2017.02.012 28351613

[B9] Guan Y. Gong Z. Xiao T. Li Z. (2018). Knockdown of miR-572 suppresses cell proliferation and promotes apoptosis in renal cell carcinoma cells by targeting the NF2/Hippo signaling pathway. Int. J. Clin. Exp. Pathol. 11 (12), 5705–5714. 31949656 PMC6963082

[B10] Guo R. Zou B. Liang Y. Bian J. Xu J. Zhou Q. (2021). LncRNA RCAT1 promotes tumor progression and metastasis via miR-214-5p/E2F2 axis in renal cell carcinoma. Cell Death Dis. 12 (7), 689. 10.1038/s41419-021-03955-7 34244473 PMC8270952

[B11] Gurova K. V. Hill J. E. Razorenova O. V. Chumakov P. M. Gudkov A. V. (2004). p53 pathway in renal cell carcinoma is repressed by a dominant mechanism. Cancer Res. 64 (6), 1951–1958. 10.1158/0008-5472.can-03-1541 15026329

[B12] Harb O. A. Elfeky M. A. El Shafaay B. S. Taha H. F. Osman G. Harera I. S. (2018). SPOP, ZEB-1 and E-cadherin expression in clear cell renal cell carcinoma (cc-RCC): clinicopathological and prognostic significance. Pathophysiology 25 (4), 335–345. 10.1016/j.pathophys.2018.05.004 29801752

[B13] Heinicke F. Zhong X. Zucknick M. Breidenbach J. Sundaram A. Y. M. S T. F. (2020a). Systematic assessment of commercially available low-input miRNA library preparation kits. RNA Biol. 17 (1), 75–86. 10.1080/15476286.2019.1667741 31559901 PMC6948978

[B14] Heinicke F. Zhong X. Zucknick M. Breidenbach J. Sundaram A. Y. M. S T. F. (2020b). An extension to: systematic assessment of commercially available low-input miRNA library preparation kits. RNA Biol. 17 (9), 1284–1292. 10.1080/15476286.2020.1761081 32436772 PMC7549702

[B15] Hill M. Tran N. (2018). MicroRNAs regulating MicroRNAs in cancer. Trends Cancer 4 (7), 465–468. 10.1016/j.trecan.2018.05.002 29937044

[B16] Hill M. Tran N. (2021). miRNA interplay: mechanisms and consequences in cancer. Dis. Model Mech. 14 (4), dmm047662. 10.1242/dmm.047662 33973623 PMC8077553

[B17] Hu H. Miao Y. R. Jia L. H. Yu Q. Y. Zhang Q. Guo A. Y. (2019). AnimalTFDB 3.0: a comprehensive resource for annotation and prediction of animal transcription factors. Nucleic Acids Res. 47 (D1), D33-D38–D8. 10.1093/nar/gky822 30204897 PMC6323978

[B18] Huang H. Y. Lin Y. C. Li J. Huang K. Y. Shrestha S. Hong H. C. (2020). miRTarBase 2020: updates to the experimentally validated microRNA-target interaction database. Nucleic Acids Res. 48 (D1), D148-D154–D54. 10.1093/nar/gkz896 31647101 PMC7145596

[B19] Huang T. Wang G. Yang L. Peng B. Wen Y. Ding G. (2017). Transcription factor YY1 modulates lung cancer progression by activating lncRNA-PVT1. DNA Cell Biol. 36 (11), 947–958. 10.1089/dna.2017.3857 28972861

[B20] Huang X. Jiang L. Lu S. Yuan M. Lin H. Li B. (2022). Overexpression of ERCC6L correlates with poor prognosis and confers malignant phenotypes of lung adenocarcinoma. Oncol. Rep. 48 (1), 131. 10.3892/or.2022.8342 35656882 PMC9204608

[B21] Hutter C. Zenklusen J. C. (2018). The cancer genome atlas: creating lasting value beyond its data. Cell 173 (2), 283–285. 10.1016/j.cell.2018.03.042 29625045

[B22] Ibing S. Michels B. E. Mosdzien M. Meyer H. R. Feuerbach L. Korner C. (2021). On the impact of batch effect correction in TCGA isomiR expression data. Nar. Cancer 3 (1), zcab007. 10.1093/narcan/zcab007 34316700 PMC8210273

[B23] Inoue J. Inazawa J. (2021). Cancer-associated miRNAs and their therapeutic potential. J. Hum. Genet. 66 (9), 937–945. 10.1038/s10038-021-00938-6 34088973

[B24] Jensen M. A. Ferretti V. Grossman R. L. Staudt L. M. (2017). The NCI Genomic Data Commons as an engine for precision medicine. Blood 130 (4), 453–459. 10.1182/blood-2017-03-735654 28600341 PMC5533202

[B25] Khotib J. Marhaeny H. D. Miatmoko A. Budiatin A. S. Ardianto C. Rahmadi M. (2023). Differentiation of osteoblasts: the links between essential transcription factors. J. Biomol. Struct. Dyn. 41 (19), 10257–10276. 10.1080/07391102.2022.2148749 36420663

[B26] Latini A. Vancheri C. Amati F. Morini E. Grelli S. Matteucci C. (2022). Expression analysis of miRNA hsa-let7b-5p in naso-oropharyngeal swabs of COVID-19 patients supports its role in regulating ACE2 and DPP4 receptors. J. Cell Mol. Med. 26 (19), 4940–4948. 10.1111/jcmm.17492 36073344 PMC9538662

[B27] Lazar C. Meganck S. Taminau J. Steenhoff D. Coletta A. Molter C. (2013). Batch effect removal methods for microarray gene expression data integration: a survey. Brief. Bioinform 14 (4), 469–490. 10.1093/bib/bbs037 22851511

[B28] Leek J. T. Scharpf R. B. Bravo H. C. Simcha D. Langmead B. Johnson W. E. (2010). Tackling the widespread and critical impact of batch effects in high-throughput data. Nat. Rev. Genet. 11 (10), 733–739. 10.1038/nrg2825 20838408 PMC3880143

[B29] Li S. Feng Z. Zhang X. Lan D. Wu Y. (2019). Up-regulation of microRNA-200c-3p inhibits invasion and migration of renal cell carcinoma cells via the SOX2-dependent Wnt/β-catenin signaling pathway. Cancer Cell Int. 19, 231. 10.1186/s12935-019-0944-5 31516388 PMC6731573

[B30] Li Y. Chen D. Su Z. Li Y. Liu J. Jin L. (2016). MicroRNA-106b functions as an oncogene in renal cell carcinoma by affecting cell proliferation, migration and apoptosis. Mol. Med. Rep. 13 (2), 1420–1426. 10.3892/mmr.2015.4656 26648244

[B31] Liu H. P. Lai H. M. Guo Z. (2021). Prostate cancer early diagnosis: circulating microRNA pairs potentially beyond single microRNAs upon 1231 serum samples. Brief. Bioinform 22 (3), bbaa111. 10.1093/bib/bbaa111 32607548

[B32] Liu K. Pan X. Peng X. Zhang C. Li H. Guan X. (2019). Associations of high expression of miR-106b-5p detected from FFPE sample with poor prognosis of RCC patients. Pathol. Res. Pract. 215 (6), 152391. 10.1016/j.prp.2019.03.019 31076282

[B33] Lv Y. Sun X. (2024). Role of miRNA in pathogenesis, diagnosis, and prognosis in hepatocellular carcinoma. Chem. Biol. Drug Des. 103 (1), e14352. 10.1111/cbdd.14352 37726253

[B34] Ma T. Zhao Y. Lu Q. Lu Y. Liu Z. Xue T. (2018). MicroRNA-30c functions as a tumor suppressor via targeting SNAI1 in esophageal squamous cell carcinoma. Biomed. Pharmacother. 98, 680–686. 10.1016/j.biopha.2017.12.095 29304493

[B35] Matthaei H. Wylie D. Lloyd M. B. Dal Molin M. Kemppainen J. Mayo S. C. (2012). miRNA biomarkers in cyst fluid augment the diagnosis and management of pancreatic cysts. Clin. Cancer Res. 18 (17), 4713–4724. 10.1158/1078-0432.CCR-12-0035 22723372 PMC3547600

[B36] Morita M. Yoneda A. Tokunoh N. Masaki T. Shirakura K. Kinoshita M. (2023). Upregulation of Robo4 expression by SMAD signaling suppresses vascular permeability and mortality in endotoxemia and COVID-19 models. Proc. Natl. Acad. Sci. U. S. A. 120 (3), e2213317120. 10.1073/pnas.2213317120 36634143 PMC9934020

[B37] Nersisyan S. Engibaryan N. Gorbonos A. Kirdey K. Makhonin A. Tonevitsky A. (2020). Potential role of cellular miRNAs in coronavirus-host interplay. PeerJ 8, e9994. 10.7717/peerj.9994 32983652 PMC7497610

[B38] Nygaard V. Rodland E. A. Hovig E. (2016). Methods that remove batch effects while retaining group differences may lead to exaggerated confidence in downstream analyses. Biostatistics 17 (1), 29–39. 10.1093/biostatistics/kxv027 26272994 PMC4679072

[B39] Peng H. Yu Y. Yu S. (2024). Re-thinking the effectiveness of batch normalization and beyond. IEEE Trans. Pattern Anal. Mach. Intell. 46 (1), 465–478. 10.1109/TPAMI.2023.3319005 37747867

[B40] Ritchie M. E. Phipson B. Wu D. Hu Y. Law C. W. Shi W. (2015). Limma powers differential expression analyses for RNA-sequencing and microarray studies. Nucleic Acids Res. 43 (7), e47. 10.1093/nar/gkv007 25605792 PMC4402510

[B41] Rozowsky J. Kitchen R. R. Park J. J. Galeev T. R. Diao J. Warrell J. (2019). exceRpt: a comprehensive analytic platform for extracellular rna profiling. Cell Syst. 8 (4), 352–357. 10.1016/j.cels.2019.03.004 30956140 PMC7079576

[B42] Saleh A. D. Cheng H. Martin S. E. Si H. Ormanoglu P. Carlson S. (2019). Integrated genomic and functional microRNA analysis identifies miR-30-5p as a tumor suppressor and potential therapeutic nanomedicine in head and neck cancer. Clin. Cancer Res. 25 (9), 2860–2873. 10.1158/1078-0432.CCR-18-0716 30723145 PMC6497577

[B43] Shang R. Lee S. Senavirathne G. Lai E. C. (2023). microRNAs in action: biogenesis, function and regulation. Nat. Rev. Genet. 24 (12), 816–833. 10.1038/s41576-023-00611-y 37380761 PMC11087887

[B44] Shukla A. Pandey H. M. Mehrotra D. (2015). “Comparative review of selection techniques in genetic algorithm,” in 2015 International Conference on Futuristic Trends on Computational Analysis and Knowledge Management (ABLAZE), Greater Noida, India, February, 2015.

[B45] Song Y. Kelava L. Kiss I. (2023). MiRNAs in lung adenocarcinoma: role, diagnosis, prognosis, and therapy. Int. J. Mol. Sci. 24 (17), 13302. 10.3390/ijms241713302 37686110 PMC10487838

[B46] Sun Y. Li L. Xing S. Pan Y. Shi Y. Zhang L. (2017). miR-503-3p induces apoptosis of lung cancer cells by regulating p21 and CDK4 expression. Cancer Biomark. 20 (4), 597–608. 10.3233/CBM-170585 28800319

[B47] Szafranska A. E. Davison T. S. John J. Cannon T. Sipos B. Maghnouj A. (2007). MicroRNA expression alterations are linked to tumorigenesis and non-neoplastic processes in pancreatic ductal adenocarcinoma. Oncogene 26 (30), 4442–4452. 10.1038/sj.onc.1210228 17237814

[B48] Tang H. Liu J. Huang J. (2022). GMFG (glia maturation factor gamma) inhibits lung cancer growth by activating p53 signaling pathway. Bioengineered 13 (4), 9284–9293. 10.1080/21655979.2022.2049958 35383531 PMC9161896

[B49] Tao T. Wang Y. Luo H. Yao L. Wang L. Wang J. (2013). Involvement of FOS-mediated miR-181b/miR-21 signalling in the progression of malignant gliomas. Eur. J. Cancer 49 (14), 3055–3063. 10.1016/j.ejca.2013.05.010 23810250

[B50] Tong Z. Cui Q. Wang J. Zhou Y. (2019). TransmiR v2.0: an updated transcription factor-microRNA regulation database. Nucleic Acids Res. 47 (D1), D253-D258–D8. 10.1093/nar/gky1023 30371815 PMC6323981

[B51] Vishnubalaji R. Shaath H. Al-Alwan M. Abdelalim E. M. Alajez N. M. (2022). Reciprocal interplays between MicroRNAs and pluripotency transcription factors in dictating stemness features in human cancers. Semin. Cancer Biol. 87, 1–16. 10.1016/j.semcancer.2022.10.007 36354097

[B52] Whalen S. Schreiber J. Noble W. S. Pollard K. S. (2022). Navigating the pitfalls of applying machine learning in genomics. Nat. Rev. Genet. 23 (3), 169–181. 10.1038/s41576-021-00434-9 34837041

[B53] Yang Y. Zhang T. Xiao R. Hao X. Zhang H. Qu H. (2020). Platform-independent approach for cancer detection from gene expression profiles of peripheral blood cells. Brief. Bioinform 21 (3), 1006–1015. 10.1093/bib/bbz027 30895303

[B54] Zeng T. Zhang W. Yu X. Liu X. Li M. Chen L. (2016). Big-data-based edge biomarkers: study on dynamical drug sensitivity and resistance in individuals. Brief. Bioinform 17 (4), 576–592. 10.1093/bib/bbv078 26411472

[B55] Zhang H. D. Jiang L. H. Hou J. C. Zhou S. Y. Zhong S. L. Zhu L. P. (2018b). Circular RNA hsa_circ_0072995 promotes breast cancer cell migration and invasion through sponge for miR-30c-2-3p. Epigenomics 10 (9), 1229–1242. 10.2217/epi-2018-0002 30182731

[B56] Zhang J. Cai M. Jiang D. Xu L. (2019). Upregulated LncRNA-CCAT1 promotes hepatocellular carcinoma progression by functioning as miR-30c-2-3p sponge. Cell Biochem. Funct. 37 (2), 84–92. 10.1002/cbf.3375 30773676

[B57] Zhang J. Raju G. S. Chang D. W. Lin S. H. Chen Z. Wu X. (2018a). Global and targeted circulating microRNA profiling of colorectal adenoma and colorectal cancer. Cancer 124 (4), 785–796. 10.1002/cncr.31062 29112225 PMC5801195

[B58] Zhang L. Ludden C. M. Cullen A. J. Tew K. D. Branco de Barros A. L. Townsend D. M. (2023). Nuclear factor kappa B expression in non-small cell lung cancer. Biomed. Pharmacother. 167, 115459. 10.1016/j.biopha.2023.115459 37716117 PMC10591792

[B59] Zhang N. Cao S. Sun R. Wang Y. Liu L. Wang W. (2022). Signal peptidase 21 suppresses cell proliferation, migration, and invasion via the PTEN-PI3K/Akt signaling pathway in lung adenocarcinoma. PeerJ 10, e14206. 10.7717/peerj.14206 36275477 PMC9583857

[B60] Zhang Q. Liu W. Zhang H. M. Xie G. Y. Miao Y. R. Xia M. (2020b). hTFtarget: a comprehensive database for regulations of human transcription factors and their targets. Genomics Proteomics Bioinforma. 18 (2), 120–128. 10.1016/j.gpb.2019.09.006 PMC7647694 32858223

[B61] Zhang Y. Parmigiani G. Johnson W. E. (2020a). ComBat-seq: batch effect adjustment for RNA-seq count data. Nar. Genom Bioinform 2 (3), lqaa078. 10.1093/nargab/lqaa078 33015620 PMC7518324

[B62] Zhang Y. Patil P. Johnson W. E. Parmigiani G. (2021). Robustifying genomic classifiers to batch effects via ensemble learning. Bioinformatics 37 (11), 1521–1527. 10.1093/bioinformatics/btaa986 33245114 PMC8485848

[B63] Zhu K. Lin J. Chen S. Xu Q. (2021). miR-9-5p promotes lung adenocarcinoma cell proliferation, migration and invasion by targeting ID4. Technol. Cancer Res. Treat. 20, 15330338211048592. 10.1177/15330338211048592 34723712 PMC8564129

[B64] Zhu Y. Chen B. Pan H. Sun L. Yu T. (2023). PLIC11 drives lung cancer progression through regulating the YY1/PIWIL4 axis. Mol. Carcinog. 62 (4), 427–437. 10.1002/mc.23496 36537719

