# Supplementary material for: In search of the ratio of miRNA expression as robust biomarkers for constructing stable diagnostic models among multi-center data
Source: Front Genet. 2024 Apr 30;15:1381917. doi: 10.3389/fgene.2024.1381917 (PMC11091382; doi:10.3389/fgene.2024.1381917)
Supplement: Supplementary file 3 [file Table7.DOCX]

Supplementary Material

# Supplementary Figures and Tables

Table S1. A collection of ratio features generated based on transcription factor-mediated indirect action relationships of miRNAs.

Table S2. Raw data of the ERRmiR features in Figure 4B.

Table S3. Raw data of the miRNA features in Figure 4C.

Table S4. Raw data of the ERRmiR features in Figure 5B.

Table S5. Raw data of the miRNA features in Figure 5C.

Table S6. Raw data of the ERRmiR features in Figure 6B.
